# Investigating the Phospholipid Effect on the Bioaccessibility of Rosmarinic Acid-Phospholipid Complex through a Dynamic Gastrointestinal in Vitro Model

**DOI:** 10.3390/pharmaceutics11040156

**Published:** 2019-04-02

**Authors:** Jiahao Huang, Peter X. Chen, Michael A. Rogers, Shawn D. Wettig

**Affiliations:** 1 School of Pharmacy, University of Waterloo, Waterloo, ON N2L3G1, Canada; jiahao.huang@uwaterloo.ca (J.H.); pchen@uoguelph.ca (P.X.C.); 2 Department of Food Science, University of Guelph, Guelph, ON N1G2W1, Canada; mroger09@uoguelph.ca; 3 Waterloo Institute for Nanotechnology, University of Waterloo, Waterloo, ON N2L3G1, Canada

**Keywords:** phospholipid complex, rosmarinic acid, bioaccessibility, dissolution, TNO gastrointestinal model, gastrointestinal simulator

## Abstract

Phyto-phospholipid complexes have been developed as a common way of improving the oral bioavailability of poorly absorbable phyto-pharmaceuticals; however, the complexation with phospholipids can induce positive or negative effects on the bioaccessibility of such plant-derived active ingredients in different parts of the gastrointestinal tract (GIT). The purpose of this study was to investigate the effects of phospholipid complexation on the bioaccessibility of a rosmarinic acid-phospholipid complex (RA-PLC) using the TNO dynamic intestinal model-1 (TIM-1). Preparation of RA-PLC was confirmed using X-ray diffraction, Fourier-transform infrared spectroscopy, partition coefficient measurement, and Caco-2 monolayer permeation test. Bioaccessibility parameters in different GIT compartments were investigated. Complexation by phospholipids reduced the bioaccessibility of RA in jejunum compartment, while maintaining the ileum bioaccessibility. The overall bioaccessibility of RA-PLC was lower than the unformulated drug, suggesting that the improved oral absorption from a previous animal study could be considered as a net result of decreased bioaccessibility overwhelmed by enhanced intestinal permeability. This study provides insights into the effects of phospholipid on the bioaccessibility of hydrophilic compounds, and analyzes them based on the relationship between bioaccessibility, membrane permeability, and bioavailability. Additionally, TIM-1 shows promise in the evaluation of dosage forms containing materials with complicated effects on bioaccessibility.

## 1. Introduction

The use of phospholipid complexation has been commonplace in food and pharmaceutical sciences since its first development in 1989, promising much in the delivery of poorly absorbed plant actives and some synthetic compounds, with less complicated preparation methods compared with many other formulations [1,2,3,4,5,6]. Many poorly soluble or permeable compounds have been formulated to be more effective, systemically, by complexing with dietary phospholipids through various types of interactions (i.e., hydrogen bonding, van der Waals forces, the hydrophobic effect) [7,8]. Of late, burgeoning interest in this technique arose due to its multi-ability to enhance dissolution of hydrophobic compounds, improve permeability of hydrophilic compounds, reduce gastrointestinal toxicity of non-steroidal anti-inflammatory drugs, and protect unstable phyto-pharmaceuticals [9,10,11,12]. These properties are mainly attributed to the amphiphilic and biocompatible nature of phospholipids, which possess a polar and a non-polar moiety in their structures and have considerable dispersing ability in both aqueous and oil media. Phospholipids, especially those containing phosphatidylcholine, have shown the ability to incorporate into cell membranes to replace cellular phospholipids and affect membrane fluidity, facilitating the absorption of co-administrated payloads [13,14,15,16].

The rosmarinic acid-phospholipid complex (RA-PLC) described in this work is a PLC formulation designed to enhance the oral bioavailability and certain therapeutic activities by improving the intestinal permeability of hydrophilic RA [10,17]. The complexation reduces the contact between RA and gastrointestinal fluids, increasing the possibility for these hydrophilic compounds to simultaneously cross the membrane barrier through the uptake of phospholipids by intestinal membrane. In a recent study, the permeability coefficient and bioavailability of RA-PLC in rats were determined to be 3.15-fold and 1.25-fold higher than unformulated RA [10].

Systemically viewing the intestinal absorption, bioaccessibility is an important factor apart from membrane permeability. A prerequisite for oral bioavailability is bioaccessibility, defined as the amount of a given compound in a form that can be readily absorbed in gastrointestinal tract (GIT) [18,19,20]. Phospholipid complexation exhibits two opposing effects on the bioaccessibility according to many studies. On the one side, when RA, or similar poorly absorbable compounds, are complexed with phospholipid, it has been observed that the aqueous solubility and dissolution rate were reduced in both acid media and alkaline phosphate buffer, although the amphiphilic structure of phospholipid was expected to enhance the dissolution [21,22,23]. Resulting from the low gel to liquid crystal transition temperature (*T*_m_), unsaturated phospholipids are difficult to formulate as fine powders under room temperature as the low Tm values and amorphous nature lead to sticky powders that are difficult to deaggregate [24,25,26]. Due to the cohesive or agglomerated state of phospholipids, the wetting of these drug-phospholipid complexes can be compromised by the decrease in effective surface area contacting digestion fluids. The poor dispersibility retards the release of payloads into aqueous fluids, thus decreasing the drug amount to be readily bioaccessible. PLC may also positively influence the bioaccessibility of RA through a protective effect in GIT. Many studies have described significant content loss of plant-derived active ingredients under gastrointestinal conditions due to instability [27,28,29,30]. RA has been reported to undergo a 0–25% intestinal degradation due to its instability in an alkaline environment [31,32]. Through complexation with phospholipids, unstable active ingredients may become less exposed to digestion fluid due to the described poor dispersibility of the phospholipids, which may lead to a decrease in degradation rate [2,33]. Herein, based on the described opposing effects, attention needs to be paid to the uncertainty in the net effect of phospholipid complexation on RA bioaccessibility. Without such data, it would be difficult to explain whether the improved oral absorption of RA is accredited to the sum effect of increased bioaccessibility plus membrane permeability, or the improved permeability overwhelming decreased bioaccessibility. Therefore, there is demand for a systemic evaluation method on the bioaccessibility of PLC dosage forms in order to assess the net effect of phospholipid. Better understanding on the mechanism of oral absorption is expected to be provided by the analysis of bioaccessibility parameters in different compartments of a dynamic GIT, and in turn guide the formulation process.

The TNO intestinal model (TIM-1) is a dynamic, multi-compartmental digestion system simulating physiological processes in the human upper GIT through the use of biorelevant media and computer-controlled hydrodynamics [34,35,36]. The physical and biochemical parameters used in the TIM-1 were determined on the basis of extensive in vivo data from both human and animal trials. This system has been widely used in the following aspects: To assess the bioaccessibility of natural extracts and nutritional products, to test the bioequivalence of a variety of drug formulations, to evaluate the gastrointestinal stability of phytochemicals, and to predict the drug–food interactions under different conditions including human fasting and fed states [37,38,39,40,41]. The validation of experimental parameters used for TIM-1 can be found in previous literature and the latest research [37,41,42,43]. To date, no attempt in the literature is traceable to have used the TIM-1 in the bioaccessibility assessment of PLC formulations, and so in this study, TIM-1 was employed to test the bioaccessibility parameters of RA-PLC and investigate the opposing effects of phospholipid in different GIT environments within a single continuous process. We intended to provide insights into the bioaccessibility change by breaking up the digestion process into individual steps and analyzing the interplay between them. This study also demonstrates a broader application of the TIM-1 system in the assessment of PLC formulations and other phospholipid-based dosage forms loaded with hydrophilic active compounds.

## 2. Materials and Methods

### 2.1. Materials

**Figure 1:**
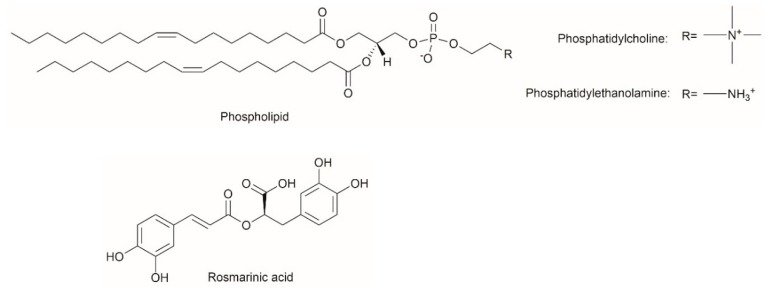
Chemical structures of phospholipid and rosmarinic acid.

RA (96%, 536954), phospholipid (P3644), 2,2-diphenyl-1-picrylhydrazyl (DPPH, D9132), and (±)-6-Hydroxy-2,5,7,8-tetramethylchromane-2-carboxylic acid (Trolox, 238813) were purchased from Sigma-Aldrich (Oakville, ON, Canada). The phospholipid used in this study contains 55% phosphatidylcholine, 25% phosphatidylethanolamine, and other phospholipids, giving an average molecular weight of 776 g/mol according to the product information sheet. Trace components in the phospholipid including triglycerides and cholesterol are not routinely quantified. ReagentPlus^®^ grade (99.5%) 1-octanol, dimethyl sulfoxide (DMSO), high-performance liquid chromatography (HPLC) grade methanol, and trichloromethane were purchased from Sigma-Aldrich (Oakville, ON, Canada). Cell culture reagents and other chemicals were supplied by Fisher Scientific (Hampton, NH, USA) and used as received. Reagents and enzymes used in the TIM-1 system including lipase, pepsin, amylase, pancreatin, trypsin, hydroxypropylmethylcellulose (HPMC), bile salts, sodium chloride, potassium chloride, calcium chloride di-hydrate, sodium bicarbonate, and hydrochloric acid were purchased from Sigma-Aldrich (Oakville, ON, Canada). Fresh porcine bile was obtained from Conestoga Meats in Breslau, ON. Water used in this study was obtained from a Millipore Milli-Q system (MilliporeSigma, Burlington, MA, USA). The chemical structures of phospholipid and rosmarinic acid are shown in Figure 1.

### 2.2. Preparation of the RA-PLC and Physical Mixture of RA and Phospholipid

RA-PLC were prepared by the solvent evaporation method. Unformulated RA and phospholipid with a molar ratio of 1:1.5 were fully dissolved (visually inspected, sonication was used as needed) in anhydrous methanol to give a final solution with a concentration of 2.5 mg RA/mL. The methanol was removed by rotary evaporation at 45 °C. The resulting mixture was solubilized with trichloromethane to obtain a solution of RA-PLC, after which the solution was centrifuged at 5000 rpm for 10 min to remove free RA. The collected supernatant was dried under reduced pressure at room temperature for 24 h to remove the residual solvent. The physical mixture (PM) of RA and phospholipid was prepared by mixing two components at the above molar ratio in a glass mortar.

### 2.3. Content Determination of RA in RA-PLC

Standard solutions of RA in methanol were prepared and determined spectrophotometrically on a plate reader (SpectraMax M5, Molecular Devices, LLC., San Jose, CA, USA) at 330 nm to give a linear Beer–Lambert calibration curve (0–15 mg/L, *r*^2^ = 0.998). A total of 15 mg RA-PLC was fully dissolved in 10 mL methanol and analyzed. The measurements were run in triplicate and the average concentration with standard deviation is reported.

### 2.4. Powder X-ray Diffraction (PXRD)

To confirm the complexation between RA and phospholipid, the physical state of RA, phospholipid, RA-PLC, and PM were analyzed on a MiniFlex X-ray diffractometer (Rigaku Corporation, Tokyo, Japan). Diffractograms were recorded over a 2θ angle from 5° to 40° at a scanning rate of 2° per minute and a 0.02° step size.

### 2.5. Fourier-Transform Infrared (IR) Spectroscopy

IR spectra of RA-PLC, unformulated RA, phospholipid, and physical mixture (PM) were recorded in transmission mode using a Perkin-Elmer RX I infrared spectrometer (Perkin-Elmer Inc, Waltham, MA, USA) equipped with diamond ATR attachment, to further validate the formation of RA-PLC. Samples were pressed into thin films without diluent and analyzed within a scan range of 4000 to 450 cm^−1^ with a resolution of 1 cm^−1^.

### 2.6. n-Octanol/Water Partition Coefficient (P) Determination

*P* values of RA-PLC and unformulated RA were determined by the shake flask method at room temperature, to compare the lipophilicity of different samples. Different media including Milli-Q water, hydrochloric acid solution (HCl, pH 1.2), and phosphate buffer solution (PBS, pH 6.8) were pre-saturated with 1-octanol before tests. Different samples equivalent to 5 mg of RA were added to sealed glass containers containing 10 mL of testing medium and stirred on a hotplate at 150 rpm for 24 h. All samples were centrifuged to obtain an aqueous phase. Afterwards, 5 mL of these aqueous samples were added to 5 mL of 1-octanol (pre-saturated with corresponding aqueous media) and stirred at 150 rpm for another 24 h. The liquid mixtures were left to separate for 24 h to obtain aqueous and organic phases. The RA concentration in aqueous phase of each step was determined spectrophotometrically at 330 nm. All measurements were run in triplicate. 

The *P* values of RA in different samples were calculated as following:

$$P=\frac{C_{1}-C_{2}}{C_{2}},$$

where *C*_1_ represents the RA concentration in the aqueous phase in the first step, and *C*_2_ represents the RA concentration in the aqueous phase after water-octanol phase separation.

### 2.7. Radical Scavenging Activity Assay

The antioxidant activities of RA and RA-PLC were assessed using an in vitro chemical model system, DPPH, to test the possible change in the bioactivity of RA induced by the co-administration of phospholipid. The antiradical activity was determined spectrophotometrically as described previously [44]. DPPH working solution (350 μM) and Trolox standard solutions (1000, 750, 500, 250, 125, and 62.5 μM) were prepared by dissolving the required amounts of each in anhydrous methanol. Samples equivalent to 5 mg of RA were dissolved in 5 mL of methanol, and 25 μL of this sample solution was mixed with 200 μL of DPPH working solution in a 96-well plate, after which the plate was sealed, and the mixture was allowed to incubate for 6 h at room temperature. Finally, the absorbance was recorded at 517 nm.

The percentage DPPH quenched (%) was determined as following:

$$\mathrm{DPPH}\;\mathrm{quenched}\;\left(\%\right)=\left[1-\frac{A_{\mathrm{sample}}-A_{\mathrm{blank}}}{A_{\mathrm{control}}-A_{\mathrm{blank}}}\right]\times 100,$$

where *A*_sample_, *A*_blank_, and *A*_control_ refer to absorbance values of the sample, methanol, and DPPH methanol solution at 517 nm. DPPH quenched (%) was plotted against the concentrations of Trolox, and the antioxidant capacity of samples were calculated based on the linear calibration curve. The antioxidant activity was expressed as mM Trolox equivalent (TE) per mM RA.

### 2.8. Cell Culture

The Caco-2 human intestine cell line obtained from Sigma Aldrich Canada was cultured in high-glucose Dulbecco’s Modified Eagle’s Medium (DMEM) supplemented with 10% fetal bovine serum and 1% penicillin–streptomycin. The cells were incubated under a 5% CO_2_ atmosphere at 37 °C and spent media was replaced every 2 days. After reaching 80–90% confluency in a T-75 culture flask, cells were harvested with 0.25% trypsin–ethylenediaminetetraacetic acid (EDTA) solution and sub-cultured to proper culture plates depending on the experiments to be conducted.

### 2.9. Cell Viability Assay

The cytotoxic effects of unformulated RA and RA-PLC on Caco-2 cells were determined using a 3-(4,5-dimethylthiazol-2-yl)-2,5-diphenyltetrazolium bromide (MTT) assay. RA and RA-PLC were dissolved or dispersed in DMEM and then diluted to different concentrations. Caco-2 cells were sub-cultured in a 96-well plate at a density of 1.0 × 10^5^ cells/cm^2^ and allowed to attach. The cells were incubated with sample solutions at RA concentrations of 10, 20, and 50 μg/mL for 4 h, and then the samples were removed and the cells were rinsed with Hank’s balanced salt solution (HBSS). The cells were treated with MTT (0.5 mg/mL) for 4 h under 5% CO_2_ at 37 ℃, after which the medium was aspirated and 100 μL of DMSO was added to each well to dissolve formazan crystals. The plate was shaken for 5 min and the absorbance was recorded at 570 nm with untreated cells as control. The cell viability (%) was calculated as following:

$$\mathrm{Cell}\;\mathrm{viability}\;\left(\%\right)=\frac{A_{\mathrm{sample}}-A_{\mathrm{blank}}}{A_{\mathrm{control}}-A_{\mathrm{blank}}}\times 100,$$

where *A*_sample_, *A*_blank_, and *A*_control_ refer to absorbance values of sample, blank media, and untreated cells, respectively.

### 2.10. Caco-2 Cell Transport Assay

Caco-2 cells were cultured and harvested as described in Section 2.9, followed by seeding in trans-well inserts (Corning Costar Corporation, Tewksbury, MA, USA) in a 6-well plate with a density of 1.0 × 10^5^ cells per cm^2^. The media in both upper and lower compartments were replaced every other day and the cells were cultivated over 21 days to achieve a confluent monolayer. RA and RA-PLC were dissolved or dispersed in HBSS buffer to a concentration of 50 μg/mL and applied onto either the apical (AP) side or basolateral (BL) side in an atmosphere of 5% CO_2_ at 37 ℃, while the recipient compartments were filled with 1 mL blank HBSS. Recipient samples were withdrawn from BL side in AP-BL test, and AP side in BL-AP test at 1, 2, 3, and 4 h after sample treatment, and then a same amount of blank solution was supplied to recipient compartment. RA concentrations of all samples were determined spectrophotometrically on a plate reader at 330 nm based on a linear Beer–Lambert calibration curve with HBSS as blank control. All experiments were run in triplicate. The apparent permeability coefficient (*P*_app_) was calculated as following:

$$P_{\mathrm{app}}=\frac{dQ/dt}{A\times C_{0}},$$

where d*Q*/d*t* (μg/s) is the rate of permeation of RA across the monolayer as described by the linear appearance rate of RA in the recipient compartment, *A* is the surface area of cell monolayer (4.2 cm^2^ in this study), and *C*_0_ is the initial RA concentration (49.10 μg/mL) in the donor compartment.

The net efflux ratio was calculated following the equation below:

$$\mathrm{Efflux}\;\mathrm{ratio}=\frac{P_{\mathrm{app}}\left(\mathrm{BL}-\mathrm{AP}\right)}{P_{\mathrm{app}}\left(\mathrm{AP}-\mathrm{BL}\right)},$$

where *P*_app_ (BL-AP) is the *P*_app_ value from basolateral side to apical side, and *P*_app_ (AP-BL) is the *P*_app_ value from apical side to basolateral side.

### 2.11. TIM-1 Study

The TNO gastrointestinal model (TIM-1) method has been described in detail [45]. Briefly, the model consists of four compartments that model the stomach, duodenum, jejunum, and ileum, with a fluid volume of 250 mL, 55 mL, 115 mL, and 115 mL, respectively. Each compartment has an inner silicon tubing surrounded by water and encased in a glass exterior. Peristaltic valves determine the transport rate of the digestate between the different compartments. Peristaltic movement is simulated by the squeezing action of the silicon tubes as dictated by the pumping of the surrounding water. The water is set at 37 °C to ensure that all compartments are kept at a physiological temperature. The rate of secretion of digestive juices in each compartment is set in accordance to predetermined physiological data [45]. The pH of the stomach was pre-set in system protocol as follows: 3.0 at 0 min, 2.2 at 10 min, 1.8 at 30 min, and 1.7 from 60min. The pH of duodenum, jejunum, and ileum were kept at 6.3, 6.5, and 7.4, respectively. All parameters are computer-controlled and a protocol for fasted state digestion of a water-soluble compound was selected for this experiment. Transit of the test formulation between compartments is automatically controlled by system sensors that detect the volume of fluids entering the compartments, as well as their solution properties (i.e., viscosity). The residual volume for the stomach compartment was set at 40 mL. For simulation of absorption of potentially available RA, the jejunal and ileal compartments are connected to semi-permeable hollow fiber membrane units (hemodialyzer cut-off of 3–5 kD). Samples equivalent to 20 mg RA were added to 240 mL of water in the stomach compartment containing 10 mL of a gastric enzyme and HPMC/bile mixture solution at pH 2. Gastric enzyme solution consists of 6000 U lipase, 1,440,000 U pepsin and 42,000 U amylase. Each experiment was terminated after 300 min. Jejunal and ileal dialyzable fractions along with the ileal efflux were collected at 15 min intervals for the first hour, at 30 min intervals in the following hour, and at 1 h intervals for the remainder of the run. All samples were run in triplicate. Secretions and enzyme solutions were prepared in accordance with TNO-Triskelion protocols.

### 2.12. HPLC Analysis

Bioaccessible fractions from TIM-1 digestion were analyzed with an Agilent 1100 series HPLC system equipped with an auto sampler, a degasser, a quaternary pump, a diode-array detector (DAD), ChemStation software, and separated on a Zorbax 300SB-C18 column (150 × 4.60 mm, 5 μm, Agilent Inc., Santa Clara, CA, USA). The binary mobile phase consisted of 0.1% trifluoroacetic acid in water (*v*/*v*) (solvent A) and 70% methanol in water (*v*/*v*) (solvent B). The solvent gradient was as follows: 0–20 min, 0–100% B; 20–25 min, 100% B; 25.5–30 min, 0% B. Injection volume was 10 μL and the flow rate was constantly kept at 1.0 mL/min for a total run time of 30 min. Peaks were monitored at 330 nm and identified by matching the retention time and UV absorption spectra with the standard RA. Quantification was done using RA standard curve generated from serial dilutions (7.8125–2000 mg/L; *r*^2^ = 0.99).

### 2.13. Statistical Analysis

All samples were run in triplicate and concentrations were expressed as the means ± standard deviation. Data were analyzed using a one factor analysis of variance (ANOVA) with the IBM SPSS Statistics program (IBM, Armonk, NY, USA).

## 3. Results

### 3.1. RA Content in RA-PLC

The RA content was determined to be 22.6 ± 1.01% (*w*/*w*) in RA-PLC, slightly lower than the weight percent of RA in the starting material blend, which was 23.7%. The result indicated that most of the RA input was complexed to the phospholipids. The complexation efficiency is likely a result of the strong molecular interactions between RA and phospholipids, as discussed below.

### 3.2. PXRD Pattern

**Figure 2:**
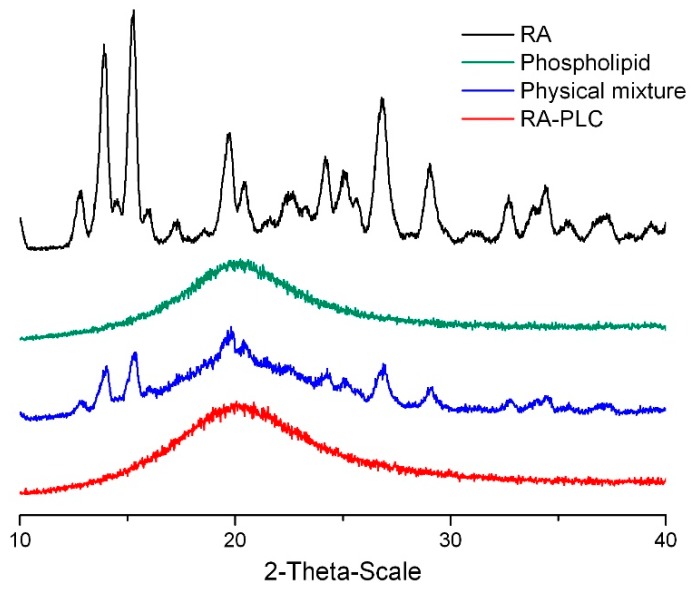
XRD diffractogram of rosmarinic acid (RA), phospholipid, physical mixture, and rosmarinic acid-phospholipid complex (RA-PLC).

PXRD patterns (Figure 2) were obtained to verify the solid state of the RA-PLC system. The diffraction pattern of unformulated RA showed a high-degree of crystallinity characterized by sharp peaks over the experimental 2θ range, as described in other literature [10]. The phospholipid used in this study showed broad amorphous bands, indicative of its non-crystalline nature. RA-PLC system appeared to be in amorphous state, as revealed by the halo bands in its spectra similar to phospholipid. This amorphous profile suggested that RA was successfully complexed to the phospholipid. In contrast to RA-PLC, the PM diffraction pattern showed a reduction on the intensity of the characteristic peaks of RA, being the sum of RA and phospholipid diffraction patterns. The maintenance of crystalline nature of RA in PM indicated that the molecular interaction between two components was limited. In conclusion, a high-degree of complexation between RA and the phospholipids in RA-PLC validated the successful preparation of a complex structure that differs from the physical mixture of two components.

### 3.3. IR Spectroscopy

**Figure 3:**
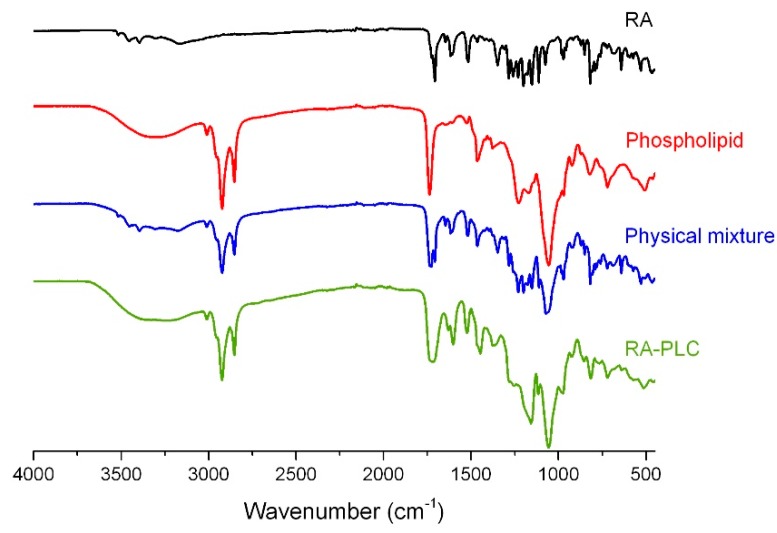
IR spectra of RA, phospholipid, physical mixture, and RA-PLC.

The IR spectra of RA, phospholipid, RA-PLC, and PM were determined to further examine the interaction between RA and phospholipid. As shown in Figure 3, the spectrum of RA showed characteristic peaks at 3518, 3454, 3396, and 3307 cm^−1^, assigned to the stretching vibration of the phenolic hydroxyl group in the structure of RA [10,46]. These peaks were also observed in the spectrum of PM, indicative of limited interactions between the phenolic hydroxyl group of RA and phospholipid structure. In contrast, these characteristic peaks were found to disappear in the spectrum of RA-PLC, suggesting that there were strong interactions between the phenolic hydroxyl of RA and the phospholipid induced by complexation. The changes likely result from the formation of hydrogen bonds between the -OH group of RA and the P=O group of the phospholipids. 

The spectrum of the phospholipids showed characteristic peaks at 2946, 2829, and 1737 cm^−1^, assigned to the non-polar saturated long-chain fatty acids of phospholipid. Similarly, these peaks were observed in the spectra of both RA-PLC and the physical mixture of RA and phospholipid, suggesting that the non-polar fatty acids did not interact with RA directly. A possible aggregation behavior of these fatty acid tails is to surround the surface of RA-PLC structure to further improve the lipophilicity of the complex. Strong interactions between RA phenolic group and phosphatidic acid group in phospholipid were considered as an evidence of a high-degree of complexation. IR spectroscopy further supported the PXRD results.

### 3.4. 1-Octanol/Water Partition Coefficient (P) of RA, RA–PLC, and PM

The P values of RA, RA-PLC, and PM determined in different media are listed in Table 1. Compared to unformulated RA, the *P* values of RA-PLC in water and phosphate buffer solution (PBS, pH 6.8) increased significantly (*p* < 0.05), attributed to the increased partitioning of RA into organic phase due to the improved lipophilicity after complexation with phospholipid. Compared with RA-PLC, PM exhibited a smaller extent of increase in *P* values in water and PBS, respectively, resulting from the slightly increased solubility of RA in 1-octanol phase induced by the in-solution interactions between RA and phospholipids. The *P* value for RA in water showed a 1.07-fold increase after physically mixing with phospholipid, and a 2.47-fold increase (*p* < 0.05) after forming RA-PLC. The difference can be explained by the different extents of interaction between RA and phospholipid as revealed by the PXRD and FTIR results. The partition of RA into the octanol phase relies on its incorporation into the amphiphilic structure of phospholipid, in favor of the RA-phospholipid interactions. Thus, compared with PM, RA-PLC showed a more effective increase in *P* value of due to the stronger interaction. A same trend can be found for PBS, where PM and RA-PLC increased the *P* value by 1.86-fold and 2.43-fold, respectively (*p* < 0.05 for both cases).

In HCl solution at pH 1.2, both RA-PLC and PM presented similar *P* values to unformulated RA. The concentrations of RA released into original aqueous phase (*C*_1_) were found to be lower than those in pure water and PBS (*p* < 0.05 in all cases). This is likely the result of poor dispersion of the phospholipid in acidic media due to protonation and electrostatic effects. When the pH of the environment is close to the first pK value of a phospholipid, intermolecular acid-anion complexation could occur through strong hydrogen bonding between the protonated phosphatidic acid (P–OH) and deprotonated phosphatidic acid (P–O^−^) groups [47,48,49]. The aggregation of phospholipids induced by intermolecular complexation was supposed to reduce the wetting of RA by incorporating a certain portion of free drug molecules and making them less exposed to aqueous media. Thus, the concentrations of RA were observed to decrease in both the first and second aqueous acid phase. As the ratio of RA concentrations in two aqueous layers, the *P* values of RA, PM, and RA-PLC showed similarity in HCl solution.

Both similarities and differences were observed when comparing our data with Yang’s results in acidic media [10]. In Yang’s study, the amount of RA in the original acidic aqueous phase was significantly decreased when physically mixing or forming complex with phospholipid, which is in agreement with our results, and can be explained by the intermolecular acid-anion complexation of phospholipids in acidic environment. However, Yang’s study showed that RA-PLC was more lipophilic than unformulated RA or the physical mixture, which is different from the results in this study. One possible reason for this difference is the composition difference in the phospholipid materials. Differentiating from the phospholipid containing 70–97% phosphatidylcholine used in Yang’s study, the one for this study contains 55% phosphatidylcholine and 25% phosphatidylethanolamine. Phosphatidylethanolamine is well characterized by its non-bulky head group with a strong tendency to form intermolecular (N–H to P–O) hydrogen bonds between the amine and phosphate group [50]. Similar to the complexation between protonated and deprotonated phosphatidic acid group, this intermolecular hydrogen bond may further facilitate the phospholipid aggregation in acidic aqueous phase. Alternatively, unlike the preparation of RA-PLC using dissolved phospholipid, the incorporation of RA into phospholipid aggregates in acidic aqueous phase can be seen as an uncontrollable process, which may or may not be accompanied by RA incorporation. When contacting octanol, the RA hosted by phospholipid aggregates can either enter the organic phase with the phospholipid aggregates or release from the aggregates to undergo partitioning individually. The experimentally determined *P* value is a final equilibrium of all these factors, as such could contribute to differences between the two studies. Given the discussed observations, future researchers are suggested to take phospholipid types and their ionization constants into consideration for the formulation work.

The increased lipophilicity of RA in water and alkaline media is expected to provide a faster partition of RA into the lipid cell membranes. Results will be discussed in Section 3.7.

### 3.5. Radical Scavenging Activity (DPPH) Assay

The major biological effect of RA is demonstrated by its ability to reduce liver damage caused by lipopolysaccharides and D-galactosamine through the scavenging of superoxide molecules [51,52,53]. As a result, the maintenance of antioxidant activity is expected to preserve the anti-inflammatory bioactivity and other pharmacological effects which are linked to degenerative and chronic diseases caused by oxidative stress [54,55]. The DPPH assay measures the reducing ability of a compound and was used in this study to assess any possible difference in the antioxidant activity between RA and RA-PLC. The TE (Trolox equivalent) values for RA and RA-PLC were calculated based on the equivalent RA content in each sample. The results were calculated to be 3.62 ± 0.08 mM and 3.64 ± 0.09 mM, respectively, suggesting that the antioxidant activity of RA was not compromised by the co-administration of phospholipid. Thus, the possible change in RA biological activity in PLC formulation is considered as the result of bioaccessibility alteration, of which the importance is emphasized in this study.

### 3.6. Cell Viability

**Figure 4:**
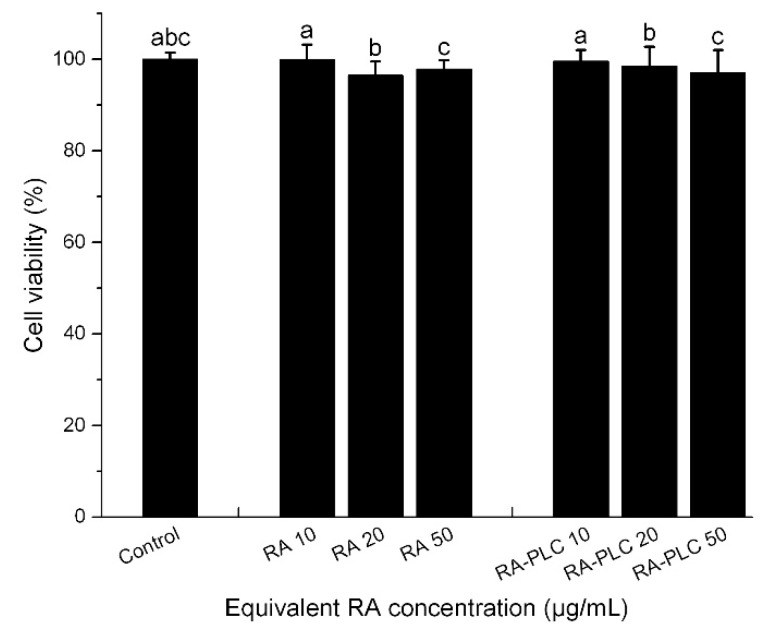
Cell viability (%) of RA and RA-PLC at different concentrations. Shared letters indicate no significant difference in cell viability between compared samples (*n* = 3, mean ± SD).

RA and RA-PLC samples equivalent to 10, 20, and 50 μg/mL RA were used to incubate Caco-2 cells to assess the effects of RA and phospholipid on cell viability. It can be seen from Figure 4 that unformulated RA did not induce obvious cell death up to a dose of 50 μg/mL. After complexation with phospholipid, the cell viability was not compromised at these concentrations. Therefore, the Caco-2 cells were treated with an equivalent RA concentration of 50 μg/mL in the following transport assay.

### 3.7. Caco-2 Transport Assay

**Figure 5:**
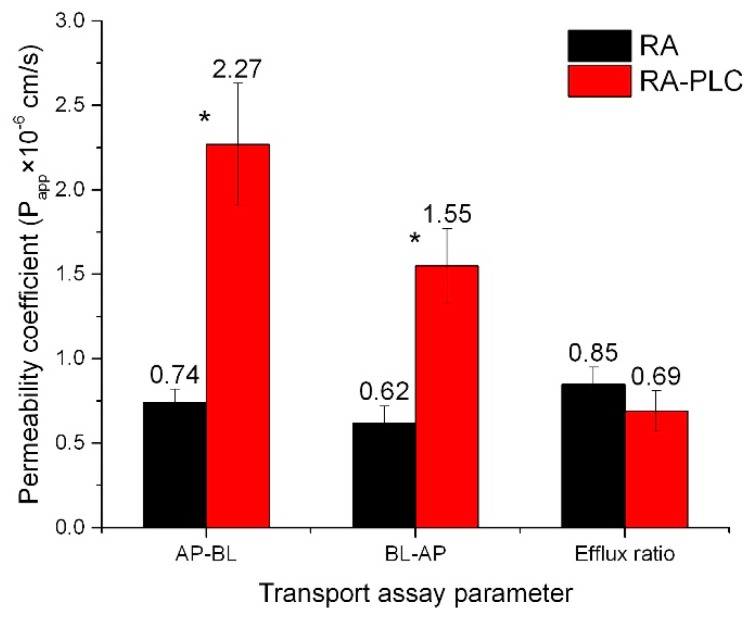
Permeability coefficient of RA and RA-PLC. * *p* < 0.05 between RA and RA-PLC (*n* = 3, mean ± SD).

As presented in Figure 5, the membrane permeability of RA was improved from both AP-BL and BL-AP sides (*p* < 0.05). The permeability coefficients were increased by 3.07-fold on AP-BL side and 2.50-fold on BL-AP side. These improvements could be attributed to the enhanced lipophilicity of RA, as revealed by the increased *P* value in phosphate buffer, and the increased uptake of RA through the simultaneous incorporation of phospholipid into cell membranes. The *P* value of RA-PLC was 0.17 in phosphate buffer, compared with that of unformulated RA, 0.07. According to Artursson’s correlation between drug lipophilicity and apparent permeability coefficients in the Caco-2 model, an increase in drug lipophilicity leads to an increased *P*_app_, as it makes the drug partition faster into the lipid cell membranes [56]. Drugs that can be completely absorbed in humans have *P*_app_ values over 1 × 10^−6^ cm/s, while those with *P*_app_ values less than 1 × 10^−8^ cm/s can only be absorbed to a value of less than 1% [56]. According to the Artursson’s correlation, the absorption of unformulated RA in human should be within the range of 50% to 100%, while RA-PLC is expected to increase the value to approximately 100%. The efflux ratio of RA-PLC was decreased to 0.69, compare with 0.85 of unformulated RA, indicating the uptake of RA through caco-2 monolayer could be effectively improved by RA-PLC. Combined with results of PXRD, FTIR, and o/w partition coefficient tests, the Caco-2 transport assay suggested a successful complexation between RA and phospholipid. RA-PLC was demonstrated to be a complex entity possessing different structure and physiochemical properties from a physical mixture. In this regard, prepared RA-PLC was assessed using the TIM-1 system in the following steps.

### 3.8. In Vitro Bioaccessibility 

#### 3.8.1. Cumulative Bioaccessibility

The bioaccessibility of RA and RA-PLC were studied through the TIM-1 system operating in a water mode, in which the bioaccessible portion was the amount of RA detected in the dialysate. The cumulative contents of bioaccessible RA in jejunum dialysate, ileum dialysate and ileum effluent were determined.

**Figure 6:**
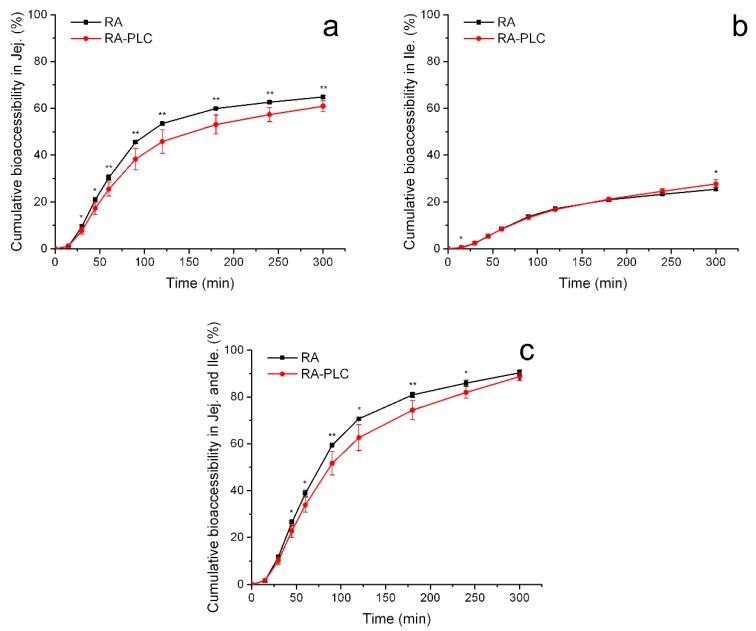
Cumulative bioaccessibility of RA and RA-PLC in jejunum (**a**), ileum (**b**), and the sum (**c**). * *p* < 0.2 and ** *p* < 0.1 between RA and RA-PLC (*n* = 3, mean ± SD).

In general, as shown in Figure 6a, the total cumulative amounts of bioaccessible RA in the jejunum compartment increased continuously for both unformulated material and RA-PLC within 5 h. Within each time interval, unformulated RA showed higher bioaccessibility compared to RA-PLC, indicating a retarded release of RA after complexing with phospholipid. At the beginning of jejunum digestion, the slower release from RA-PLC could be explained by a gastric effect from the previous digestion step (i.e., within the stomach compartment). The protonation of the phospholipids in gastric fluid could induce an intermolecular acid-anion complexation as discussed in Section 3.4, resulting in poorly dispersible state for the phospholipid. A certain portion of RA was both complexed with phospholipid molecules and surrounded by the phospholipid aggregate, thus the contact between RA and aqueous media was reduced. Therefore, a smaller amount of RA was released from RA-PLC into aqueous media and transported from stomach to jejunum in a dissolved form that could be detected directly. This observation corresponds well to the findings from the partition coefficient tests, which showed a reduced RA concentration from RA-PLC in acidic media. With the continuous processing of jejunum digestion, the protonation of phospholipids in the gastric environment became less of a determinant in bioaccessibility as the phospholipids could disperse well in alkaline media. The bioaccessibility difference between RA and RA-PLC became smaller gradually, indicative of similar release behaviors for the two samples in alkaline environment. Finally, the maximum bioaccessibility of RA in jejunum compartment within 5 h was determined to be 64.9% of the input amount, slightly higher than the 60.9% of RA-PLC.

In the ileum compartment, as shown in Figure 6b, the digestion of both RA and RA-PLC showed a monotonic increase in bioaccessibility, similar to that observed in the jejunum chamber. Within most time intervals, no significant difference was found between the bioaccessibility of RA and RA-PLC, indicative of similar dissolution behaviors of two samples. An interesting difference between RA and RA-PLC was observed in the profile obtained for the ileum compartment after 4 h, where the cumulative bioaccessibility of RA-PLC became higher than unformulated RA, which suggested the dissolution of RA was enhanced in the presence of phospholipids. In contrast to the bioaccessibility relation between RA and RA-PLC in previous chambers, the release rate of RA from the complex was enhanced by the amphiphilic nature and fine dispersible state of phospholipid in the alkaline intestinal environment, and by virtue of the long exposure time in the intestinal tract. After 5 h digestion, the maximum bioaccessibility of RA-PLC in the ileum compartment was determined to be approximately 28%, slightly higher than the 25% obtained for unformulated RA.

The overall bioaccessibility was defined as the sum of the amount of bioaccessible RA in both the jejunum and ileum dialysates. As shown in Figure 6c, the total bioaccessibility of unformulated RA was higher than RA-PLC for every time interval; however, it should be noticed that the bioaccessibility gap between RA and RA-PLC decreased at longer times, due to the enhancement of bioaccessibility seen for the RA-PLC in the ileum chamber at long times. At the end of the digestion process, the bioaccessibility of unformulated RA was ~90%, essentially equivalent to the ~89% obtained for RA-PLC. A more sustained digestion profile of RA-PLC was shown in Figure 6c.

#### 3.8.2. Non-Cumulative Bioaccessibility

Non-cumulative bioaccessibility of RA and RA-PLC was studied to analyze the digestion behavior of RA in each time interval, further supporting the findings from cumulative bioaccessibility study. Bioaccessibility rate was defined as the amount of RA becoming bioaccessible per minute and was plotted against digestion time.

**Figure 7:**
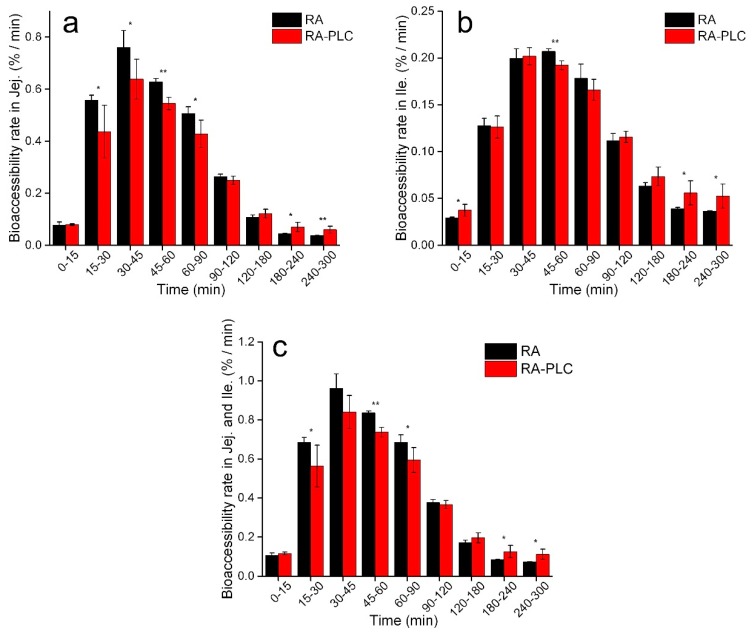
Bioaccessibility rate of RA and RA-PLC in jejunum (**a**), ileum (**b**), and the sum (**c**). * *p* < 0.2 and ** *p* < 0.1 between RA and RA-PLC (*n* = 3, mean ± SD).

From Figure 7a, it was observed that the bioaccessibility rate of unformulated RA was higher than for RA-PLC in the jejunum up to 120 min. Within this period, the bioaccessibility rate for unformulated RA rose from 0.08%/min to 0.76%/min and then decreased to a value of 0.26%/min for the 90–120 min timepoint, while the rate of RA-PLC increased from 0.08%/min to the peak of 0.64%/min and then decreased to 0.25% for the 90–120 min timepoint. By virtue of the ready dissolution of unformulated RA in gastric fluid, a larger amount of RA could be transported to the jejunum compartment in a dissolved state to be instantly detected as bioaccessible, showing as a burst release profile for RA in the initial stage of jejunum digestion. In contrast, the lower peak value of bioaccessibility rate of RA-PLC (i.e., a slower rate of increase for bioaccessibility of RA from RA-PLC) could be explained by the poor dispersing behavior of the phospholipids in gastric fluid as discussed above. With less effective wetting process in the stomach, a smaller amount of free RA could be transported to jejunum in a dissolved state to be immediately determined.

At 90 min, the bioaccessibility rate of unformulated RA decreased significantly and the difference in bioaccessibility rate between RA and RA-PLC became non-significant due to two reasons. First, most of the RA had already been dissolved and was bioaccessible, and/or transported to the ileum compartment for the next step of digestion, decreasing the following bioaccessibility rate for RA. Second, the dissolution of RA-PLC was maintained at a higher level in the jejunum as compared to RA by virtue of the exposure of phospholipid to the alkaline environment (again as described above). After 120 min timepoint, the bioaccessibility rate of RA-PLC became higher than RA. The more sustained bioaccessibility rate profile for RA-PLC from 90 min to 300 min is attributed to the continuous RA release from the reservoir that formed in stomach due to phospholipid complex aggregation. The absolute value of bioaccessibility rate of RA-PLC became larger than that of RA from 120 min, further supporting the discussion.

During ileum digestion, comparable profiles were observed in the bioaccessibility rate of RA and RA-PLC, especially at longer times. According to Figure 7b, the bioaccessibility rate of unformulated RA increased to peak within 60 min and started to decrease significantly thereafter. As a comparison, the rate of RA-PLC increased to maximum in 45 min, presenting a shorter time required to reach maximum. The results suggested that ileum environment is a dissolution-favored environment more for RA-PLC than RA, likely resulting from the amphiphilicity of phospholipid which facilitates the wetting of RA. Compared to unformulated RA, a more sustained bioaccessibility rate profile of RA-PLC was observed similar to that in jejunum compartment. The continuous release of RA from RA-PLC was likely a result of the extra time required for RA to transit from the jejunum into ileum compartment due to the delay in release from the stomach. Another reason could be the continuous separation of RA from the complex which still remained after a long-time digestion.

The total absorption rate was calculated in the same fashion as the total cumulative bioaccessibility, namely by summing the rates of the jejunum and ileum compartments. The total digestion rate is shown to be time-dependent as described above. As shown in Figure 7c, the overall bioaccessibility rate of RA was higher than RA-PLC in the initial stages of digestion, before 90 min, followed by a transition stage where two samples showed comparable rates (90–120 min), after which the digestion of RA-PLC reached an approximate steady state and the bioaccessibility rate became higher than for unformulated RA.

From the profiles of cumulative bioaccessibility, it can be concluded that the bioaccessibility of RA decreased at each time point due to the complexing with phospholipids, or extra time was required for RA-PLC to be bioaccessible. Not only does the analysis on the bioaccessibility rate support the above conclusion, but it also reveals differences that are more detailed, including the starting timepoint for RA-PLC to become more effective than RA, and the quantification of difference in instantaneous performance (i.e., the bioaccessibility rate for a 15-min period) between RA and RA-PLC. These details are expected to benefit the future formulation design of PLCs when modified or sustained release is needed. Besides, with the evaluation on the dispersibility, protonation, and the change in bioaccessibility rate of phospholipid complex in different digestive environments, controlled release of active ingredients to specific GIT regions may be achieved by adjusting phospholipids with different gel to liquid transition temperatures and pK values, as well as drug–phospholipid ratios.

It should be noted that for a BCS class 3 compound with high solubility and low permeability such as RA, bioaccessibility is a necessary but insufficient parameter to determine its bioavailability as the permeation of dissolved drug is the key determinant. Thus, the bioaccessibility data obtained from TIM-1 cannot be quantitatively used to predict drug absorption of this type of compounds. Even if combining with a caco-2 permeation experiment, the prediction of drug absorption may also be inaccurate when there is no quantitative translation model. Nevertheless, TIM-1 can be effectively used to reveal any unfavorable change in drug bioaccessibility induced by excipients, and warns formulation scientists of how much drug loses its potential to permeate into blood circulation. Considering the cost and ethical issues related to animal studies, TIM-1 bioaccessibility study is expected to help select the most worthwhile formulations to move into animal pharmacokinetic studies, in the best effort to improve the success possibility of drug product development.

At this point, it is observed that the RA-PLC dosage form increased the permeability of RA by 3.07-fold and decreased the bioaccessibility by only 1.8%, which is likely to correspond to the increased *C*_max_, AUC and the shorten *T*_max_ in the previous animal study [10]. These results suggest that the bioaccessibility disadvantage of RA-PLC was likely overwhelmed by its permeability enhancement. When formulating phospholipid complexes, it is suggested to conduct a parallel comparison between formulations using bioaccessibility change as indicator before entering animal trial, in order not to overwhelm the permeation enhancement by the decreased bioaccessibility. Several factors were identified as important considerations for future research, including ionization constant, intermolecular complexation of phospholipids, intestinal transition time, and pH changes. Currently, however, there is no way to correlate these factors with pharmacokinetic profiles in a quantitative manner. Here, we do not suppose that the TIM-1 system is able to perfectly mimic the real in vivo situation, instead it is an effective tool to compare formulations in parallel by breaking the digestion process into different stages and testing the complicated effects of excipients like phospholipids.

## 4. Conclusions

The effect of complexing RA with phospholipids on the RA bioaccessibility was successfully evaluated using the TIM-1 dynamic system where the net effect was shown to be a slight reduction in bioaccessibility for RA-PLC (88.7%) compared to unformulated RA (90.4%). The bioaccessibility profiles of RA-PLC were shown to be dependent on different digestive environments. The complexation with phospholipids decreased the bioaccessibility of RA in the early stage of jejunum digestion, while providing a more sustained digestion profile in the following ileum process. The reduction of jejunum bioaccessibility was considered as a result of poor dispersion of phospholipids in the stomach. As the prerequisite for oral bioavailability, these bioaccessibility profiles are expected to provide rational predictions on the absorption behaviors of tested formulations. In this regard, the improved oral bioavailability of RA in rats from previous research could be considered as a net result of significantly increased intestinal permeability and slightly decreased bioaccessibility.

The insights into the pH-dependent effects of phospholipid materials on the bioaccessibility of a hydrophilic compound acknowledged a potential broader application of the TIM-1 to the characterization of PLC formulations, as well as other types of dosage forms containing components with opposing effects on bioaccessibility. The study on both cumulative and non-cumulative bioaccessibility is expected to benefit the design of controlled-release PLC formulations. The combination of TIM-1 dynamic system and Caco-2 transport assay is expected to provide an alternative approach to better select formulations of low-permeable drug before moving into animal studies.

## Figures and Tables

**Table 1:** 1-Octanol/water partition coefficient of RA, physical mixture (PM), and RA–PLC in different aqueous phases (*n* = 3, mean ± SD).

Sample	Media	Concentration in Original Aqueous Phase (*C*_1_) (μg/mL)	Concentration in Separated Aqueous Phase (*C*_2_) (μg/mL)	Partition Coefficient (*C*_1_ − *C*_2_)/*C*_2_
RA	Millie Q water	488 ± 8	139 ± 3	2.50 ± 0.06
	HCl (pH 1.2)	500 ± 10	4.91 ± 0.09	100 ± 4
	PBS (pH 6.8)	483 ± 3	452 ± 4	0.068+0.005
PM	Millie Q water	500 ± 4	136 ± 4	2.68 ± 0.09
	HCl (pH 1.2)	350 ± 8	3.5 ± 0.3	100 ± 10
	PBS (pH 6.8)	491 ± 8	433 ± 6	0.131 ± 0.004
RA-PLC	Millie Q water	500 ± 7	70 ± 3	6.2 ± 0.4
	HCl (pH 1.2)	325 ± 7	3.25 ± 0.06	99 ± 3
	PBS (pH 6.8)	493 ± 6	423 ± 7	0.165 ± 0.005
